# Characterization of a P1-like bacteriophage carrying CTX-M-27 in *Salmonella spp.* resistant to third generation cephalosporins isolated from pork in China

**DOI:** 10.1038/srep40710

**Published:** 2017-01-18

**Authors:** Ling Yang, Wan Li, Gui-Ze Jiang, Wen-Hui Zhang, Huan-Zhong Ding, Ya-Hong Liu, Zhen-Ling Zeng, Hong-Xia Jiang

**Affiliations:** 1 National Risk Assessment laboratory for antimicrobial resistance of animal original bacteria, College of Veterinary Medicine, South China Agricultural University (SCAU), Guangzhou, China; 2 Guangdong Provincial Key Laboratory of Veterinary Pharmaceutics Development and Safety Evaluation, College of Veterinary Medicine, South China Agricultural University (SCAU), Guangzhou, China

## Abstract

The aim of this study was to elucidate the epidemiology of third generation cephalosporin resistant *Samonella* isolates from pork of a slaughterhouse in China and the features of transferable elements carrying *bla*_CTX-M_ genes. One hundred and twenty-six (7.3%) *Salmonella* isolates were identified; *S*. Derby and *S*. Rissen were the most two prevalent serotypes. Among these isolates 20 (15.8%) were resistant to third generation cephalosporins and nine of them carried *bla*_CTX-M-27_. S1-PFGE and replicon typing of *bla*_CTX-M-27_-carrying plasmids showed that seven were untypeable plasmids of about 104 Kb and two were IncP plasmids of about 300 Kb. Complete sequence analysis of one PBRT-untypeable plasmid showed it was a P1-like bateriophage, named SJ46, which contained a non-phage-associated region with several mobile elements, including Tn1721, IS*Ecp1B* and IS903D. The other six 104 Kb PBRT-untypeable *bla*_CTX-M-27_-carrying plasmids also harboured the same phage-insertion region of SJ46 suggesting that they were the same P1-like bacteriophage. PFGE profiles of the parental strains revealed both potential vertical and horizontal spread of this P1-like *bla*_CTX-M-27_-containing element. Additionally, the representative gene of the P1 family bacteriophage, *repL*, was detected in 19.0% (24/126) of the isolates. This study indicated a potential role of P1-family bacteriophage in capture and spread of antimicrobial resistance in pathogens.

*Salmonella* is one of the most important causes of foodborne infections worldwide^1^. *Salmonella* has the ability to colonize the guts of healthy pigs, which can serve as carriers of this organism. When admitted to slaughterhouses, asymptomatic pigs are a potential risk for *Salmonella* contamination of pork meat and for *Salmonella* infections in human^2^.

For the treatment of salmonellosis, third generation cephalosporins (3GCs) are frequently used in human and veterinary medicine^3^. The emergence of *Salmonella spp.* isolates resistant to these drugs is of increasing public health concern^4^,^5^. Resistance to cephalosporins is principally by acquisition of plasmid carrying extended-spectrum β-lactamase (ESBL) genes, the most prevalent of which are the CTX-M-type ESBLs^6^. The increasing identification of clinical isolates containing ESBLs have led to a growing interest in researching the genetic elements responsible for their emergence and dissemination. Plasmids are considered as the most common and important element to spread ESBLs between bacterial strains. The mobilization or transfer of antimicrobial resistance gene by bacteriophages has been documented for various bacterial species, including the transfer of erythromycin resistance genes in *Streptococcus pyogenes* and *Clostridium difficile*^7^,^8^, and the transduction of β-lactamase genes in *E. coli* and *Salmonella*^9^,^10^. The horizontal spread of antimicrobial resistance genes mediated by phages and is now thought to be much more frequent than previously believed^11^,^12^. Bacteriophages have the ability to shape the bacterial microbiome in any environment. Through specialized or generalized transduction, bacteriophages can transfer genes that are advantageous to their microbial hosts, in turn promoting their own survival and dissemination^13^.

Bacteriophage P1, which was isolated in 1951 by Luigi Bertani, infects and lysogenizes *Escherichia coli* and several other enteric bacteria as independent low-copy-number plasmid-like elements^14^. The acquisition of the ESBL gene *bla*_SHV-2_ by a P1-family bacteriophage have been characterized in *E. coli*, however, there is no P1-like phage carrying *bla*_CTX-M_-type ESBL genes reported in clinical isolates of *Salmonella*^11^. In this study, we estimated the contamination of pork meat by *Salmonella* and reported the discovery of the transduction of *bla*_CTX-M-27_ gene by a P1-like bacteriophage in *Salmonella* isolated from pork from a slaughterhouse in China.

## Results

### Prevalence of *Salmonella spp.* in pork of slaughterhouse

A total of 126 (7.3%) *Salmonella* strains were isolated from 1728 pork samples from a large-scale slaughterhouse in Guangdong, China, during the period of April 2013 to April 2014. Three main serotypes were identified: *S*. Derby (66.7%, 84/126), *S*. Rissen (19.8%, 25/126) and *S*. Indiana (11.1%, 14/126). Three (2.4%) isolates could only be identified as *Salmonella* belonging to serotype of group B which can agglutinate with O-antiserum factor 4, however they were not *Salmonella* I 4, [5], 12:i-.

### Resistance to cephalosporins and ESBL gene detection

**Figure 1:**
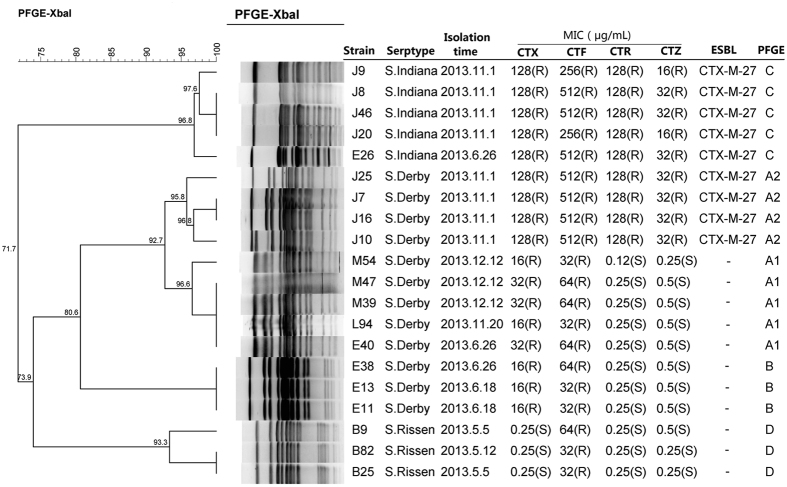
PFGE profiles of twenty 3GC-resistant *Salmonella* isolated from pork in slaughterhouse in China. MIC, minimal inhibitory concentration; CTX, cefotaxime; CTF, ceftiofur; CTR, ceftriazone; CTZ, ceftazidime; ESBL, extended-spectrum β-lactamase; PFGE, pulsed-field gel electrophoresis. “-”, not detected; “R”, resistant; “S”, susceptible.

Among the 126 *Salmonella* isolates tested, 20 (15.8%) were resistant to third-generation cephalosporins (3GCs). The resistance rate of ceftiofur, cefotaxime, ceftriaxone and ceftazidime was 15.8%, 13.5%, 7.14% and 7.14%, respectively (Fig. 1).

Among the twenty 3GCs-resistant isolates, nine (strain J7, J8, J9, J10, J16, J20, J25, J46 and E26) contained ESBL-encoding gene *bla*_CTX-M-27_. No other ESBL genes were detected, which was consistent with the results of double disk synergy test. The 9 *bla*_CTX-M-27_-positive strains were highly resistant to all four tested drugs except for ceftazidime (only 1-fold higher than the resistance break point [16 ug/mL] of ceftazidime). It is noteworthy that all *bla*_CTX-M-27_ positive strains were recovered from pork samples collected on the same day, and 5 of them were *S*. Indiana and 4 were *S*. Derby. Surprisingly, the remaining 11(8 *S*. Derby and 3 *S*. Rissen) 3GCs-resistant isolates had none of the ESBL-encoding genes tested and did not produce phenotypically ESBLs. All of the 11 non-ESBL-producing 3GCs-resistant strains were susceptible to ceftazidime and ceftriazone, but resistant to ceftiofur. Eight (72.7%) of them were resistant to both ceftaxime and cetiofur. However, their resistant levels to ceftaxime and cetiofur were lower than those of strains producing CTX-M-27 (Fig. 1).

### Pulsed-Field Gel Electrophoresis (PFGE)

The twenty 3GC-resistant *Salmonella* isolates were grouped into four XbaI-PFGE clusters designated A, B, C and D (Fig. 1). All isolates in each cluster had the same serotype. Cluster A harboured 9 *S*. Derby strains (92.7% genetic similarity). In addition, cluster A could be subgrouped into cluster A1 and cluster A2. Cluster A1 (95.8% genetic similarity) harboured five isolates, while cluster A2 (95.8% genetic similarity) was represented by four isolates containing *bla*_CTX-M-27_. Cluster B harboured three *S*. Derby strains. Cluster C harboured five *S*. Indiana containing *bla*_CTX-M-27_. Cluster D harboured three *S*. Rissen (93.3% genetic similarity).

### Sizes and replicon types of plasmids carrying *bla*_CTX-M_ gene

All plasmids carrying *bla*_CTX-M-27_ did not transfer by conjugation. Nine transformants bearing *bla*_CTX-M-27_ positive plasmid were obtained by electroporation. S1-PFGE and southern blot hybridization experiments confirmed that *bla*_CTX-M_ were located on plasmids with two different sizes, which were about 104 Kb (n = 7, strain J7, J8, J16, J20, J25, J46 and E26) and 300 Kb (n = 2, strain J9 and J10), respectively (see Fig. S1 in the Supplemental Material).

Plasmid replicon typing revealed that both ~300 Kb plasmids were of IncP type. Surprisingly, the replicon types of the remaining seven ~104 Kb plasmids could not be determined by PCR-based replicon typing (PBRT).

### Sequence analysis of a *bla*_CTX-M-27_ carrying plasmid

**Figure 2:**
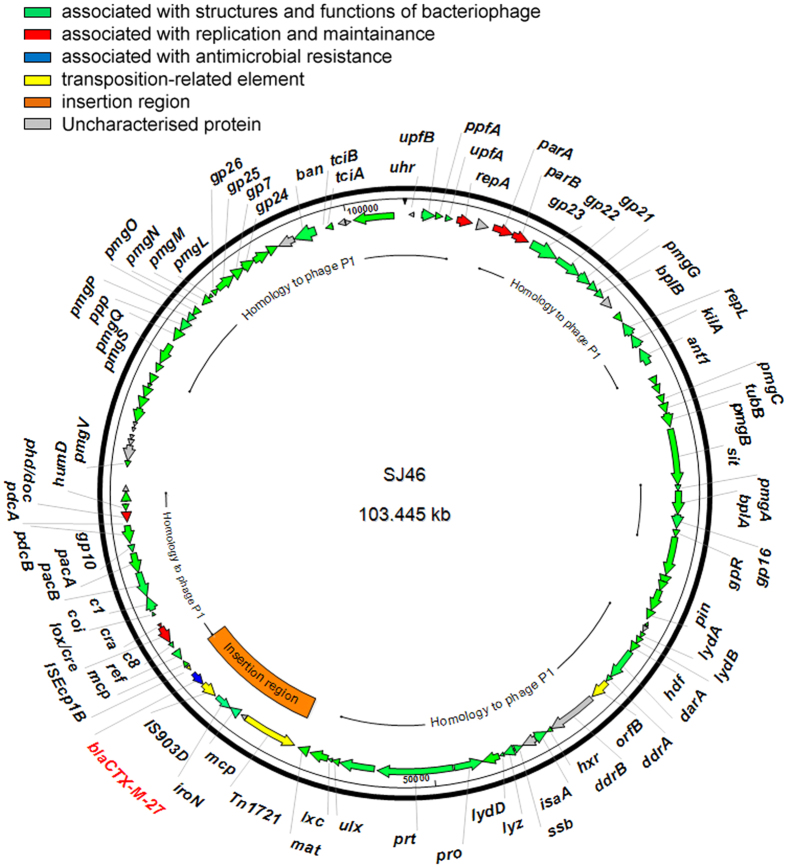
Circular map of SJ46. The coloured arrows represent ORFs and their direction of transcription.

Gene sequencing and assembling of the plasmid from strain J46 revealed a 103,445 bp plasmid, named SJ46 (GenBank acceession no. KU760857). The G + C content of SJ46 is 48.59%, and was predicted to harbor 122 protein coding sequences (Table S1) and three tRNA for asparagine, threonine and methionine. Protein-coding genes account for 90% of SJ46. The results of gene annotations revealed that SJ46 turned out to be a P1-like bacteriophage, containing genes associated with DNA replication (*repA*), partition (*parA* and *parB*), recombination (*lox/cre, cra, ref, cin*), putative morphogenetic function (*pmgA, pmgB, pmgC, pmgG, pmg*L, *pmgM, pmgN, pmgO, pmgP, pmgQ, pmgS, pmgV*), cell lysis (*repL, lydA, lydB, lydD, lyz, c1, coi, lxc*), phage structural protein (*gpR, gp7, gp16, gp21, gp22, gp23, gp24, gp25, gp26, tubB, bplA, bplB, prt, pro, mat*) and DNA packaging protein (*pacA* and *pacB*) of P1 bacteriophage and genes of other function that have been reported in P1 bacteriophage genome (Fig. 2 and Table S1)^15^.

**Figure 3:**
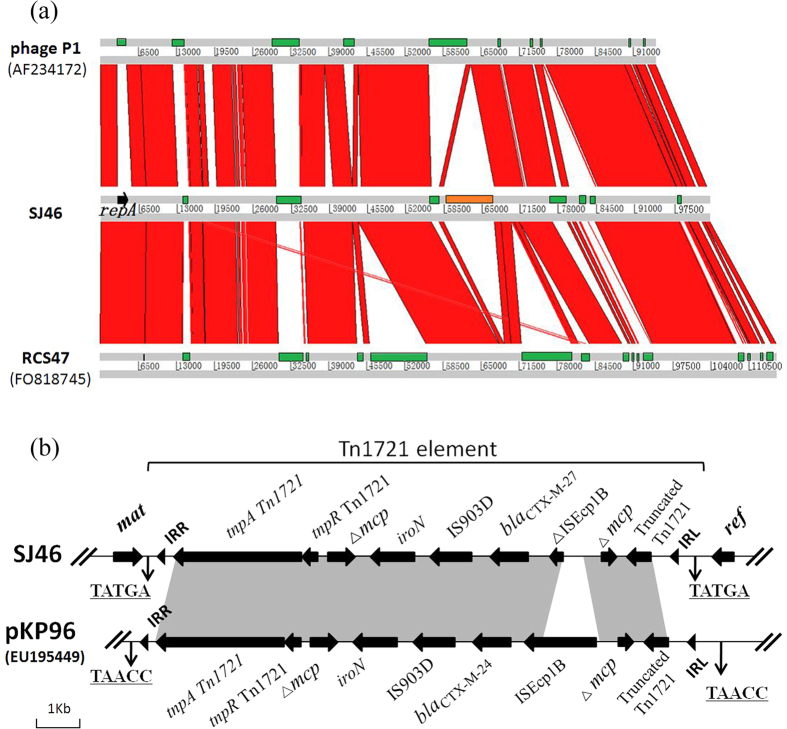
(**a**) Sequence synteny comparisons between SJ46, bacteriophage P1 (AF234172), and bacteriophage RCS47 (FO818745) as determined with the Artemis comparison tool. The green rectangles are specific genomic regions. Strand conservations are showed in red and the inserted region is indicated in orange. (**b**) Gene environment representation of *bla*_CTX-M-27_ in SJ46 and gene structure comparison of the *bla*_CTX-M_-containing region of SJ46 and pKP96. Black arrows represent ORFs and their direction. IRs of the respective mobile elements are shown as black triangles. Nucleotide letters with underlining represent a direct duplication (DR).

BLAST comparisons of the protein sequences against the GenBank databases showed that SJ46 displayed a high level identity to the P1 bacteriophage genome (GenBank accession no. AF234172.1) and P1-like bancterophage RCS47 carrying SHV-2 (GenBank accession no. FO818745.1). We found that 72% of SJ46 was common to the sequence of phage P1 bacteriophage, with the shared sequences being 98% identical and that 78% was common to the P1-like bacteriophage RCS47 carrying *bla*_SHV-2_, with the shared sequences being 99% identical (Fig. 3a). P1 bacteriophages lysogenize their hosts as autonomous plasmid-like elements and the circle format of the SJ46 was represented in Fig. 2.

SJ46 has acquired an insertion of foreign DNA, which contains a 8,644-bp *bla*_CTX-M-27_-containing region (Fig. 3a and b). It is flanked by two 5 bp direct repeat sequences (TATGA), which indicated the occurrence of a transposition event. The left inverted repeat (IRL) and the right inverted repeat (IRR) are present at the junction between inserted DNA and phage DNA. The inserted fragment containing disrupted IS*EcpIB, bla*_CTX-M-27_, IS903D and a putative iron outer membrane receptor gene *iroN* interrupts the methyl-accepting chemotaxis gene *mcp* in Tn1721. This structure was inserted between a phage particle maturation gene (*mat*) upstream and a recombination enhancement gene (*ref*) downstream. The 8.6 Kb region is similar to a *bla*_CTX-M-24_ containing region on plasmid pKP96 in *Klebsiella pneumonia* from a sputum specimen in the First Affiliated Hospital, College of Medicine, Zhejiang University in China in 2002^16^ (Fig. 3b).

### Prevalence of P1-like bacteriophage in *Salmonella* isolates

We detected *repL*, the lytic replication gene of P1 family phage, in 24 of the 126 *Salmonella* isolates from pork in this study. The gene was also detected both in seven parental strains producing CTX-M-27 (strain E26, J7, J8, J16, J20, J25 and J46) and their transformants.

S1-PFGE and southern blot assay revealed that the sizes of the *bla*_CTX-M-27_ plasmids contained in these seven strains were the same, then we assumed that these mobile elements were similar to SJ46. Thus, the presence of phage sequences in the parental strain and transformants was confirmed by specific PCR using primers at the both ends of the phage-like region where one primer is in the phage-like region and the other is in the insertion resistance region. The results showed that all seven ~104 Kb *bla*_CTX-M-27_-carrying plasmids contained the phage-insertion region structure of SJ46, which suggested that they might be the same. Interestingly, *bla*_CTX-M-27_-carrying P1-like bacteriophage was evidenced in three *Salmonella* Indiana strains (J8, J20 and J46) and three *Salmonella* Derby strains (J7, J16 and J25) isolated from samples collected on the same day (November 1^st^, 2013). According to their PFGE profiles the three *Salmonella* Indiana strains were genetically similar as were two of the three *Salmonella* Derby suggesting, on one hand clonal spread of strains carrying *bla*_CTX-M-27_-P1-like bacteriophage and, on the other hand horizontal transfer of the *bla*_CTX-M-27_-P1-like bacteriophage in *Salmonella* strains of different serotypes.

## Discussion

In addition to the impact of economic losses due to the salmonellosis for the swine industry, it is also a potential threat to human health. Several studies have indicated the role that the slaughter process can play in the spread and dissemination of *Salmonella*^17^. In the present study, *Salmonella* were detected in 7.3% (126/1728) of all pork samples. This was lower than the 68.9% reported by Y. Li *et al*. for swab samples from pigs after evisceration in slaughterhouses in Yangzhou, China^18^. This value is also lower than that of fresh slaughtered chicken meat from Shanxi (30%), Jiangsu (37.5%) and Guangdong (43.3%), China^19^.

*S*. Derby strains have been frequently isolated from pigs and pork in other countries, such as the UK^20^, France and Europe^21^. It was worrying that *S*. Derby was also the most common serotype isolated from infants and pre-school age children in China^22^. It was noteworthy that *Salmonell*a Typhimurium or Enteritidis were always detected as the second or third most prevalent serotype in China. However, in our study, *S*. Rissen was the second prevalent serotype. Globally, *S*. Rissen is an infrequently reported serotype, but it is among the top three serotypes found in pigs and pork products in Spain and in some Southeast Asian countries^23^,^24^. *S*. Rissen was also recognised as a pathogen in outbreaks of human salmonellosis in Italy and USA^25^,^26^. The prevalence of *S*. Rissen in pork is of concern because it has been responsible for sporadic human infections in China^22^. This is the first report about the prevalence of *S*. Rissen in pork meat from China.

Nearly 16% (20/126) of isolates exhibited resistance to third-generation cephalosporins. Of these, 45% (9/20) were positive for CTX-M-27 β-lactamase and no other ESBL-encoding genes were detected. CTX-M-65, CTX-M-55 and CTX-M-14 used to be the most prevalent ESBLs detected in *E. coli* from food-producing animals, but CTX-M-27 was only sporadically detected in *E. coli*^27^. There is little literature reporting the prevalence of CTX-M-27 in *Salmonella* strains in China until recent times when a study detected *bla*_CTX-M-27_ as the most dominant ESBL genes in *Salmonella* Typhimirium and Indiana isolated from pigs and Chicken^28^. The high prevalence of *bla*_CTX-M-27_ in *Salmonella* from pork meat should be a cause of concern which will be a potential threat to public health.

Eleven (55%) of the 20 3GCs-resistant *Salmonella* isolates harboured no ESBLs genes. All of them were resistant to cetiofur and eight of them to ceftaxime. These data suggested that 3GCs resistance in these strains was mediated by other mechanisms, such as the absence of out membrane proteins (eg. OmpC and OmpF) and overexpression of efflux pump AcrAB-TolC. In *Salmonella*, OmpF and OmpC are the major porins, and AcrB is also the major, constitutive multidrug pump^29^,^30^. Most cephalosporins and penicillins are substrates of AcrB^30^. It has been reported that active efflux cannot increase the MICs of β-lactams (such as ampicillin and cephalothin) in *E. coli*, however, if porin permeability is decreased, efflux would produce a more visible effect^31^. Interestingly, one study showed that mutants lacking the wider-channel OmpF and producing only the more restrictive OmpC porin become much more resistant to good substrates of AcrB, such as benzylpenicillin or cephaloram, but not to poor substrates of AcrB, such as cefazolin and cephaloridine^31^. Laboratory selection using ceftazidime or ceftibuten, starting from an *E. coli* strain containing a TEM-1-producing plasmid, also resulted in the loss of OmpF or OmpF and OmpC^32^. The absence of OmpF was also seen in a ceftazidime-resistant *E. coli* strain^33^. However, changes in porins and efflux have been rarely reported for β-lactam-resistant *Salmonella* isolates. The present study did not show the roles of outer membrane proteins and efflux pumps in cephalosporin resistance in *Salmonella* isolates, however, it provides basis of further research.

Susceptible bacteria may develop resistance to antibiotics through multiple and complex mechanisms, such as mutation and horizontal gene transfer. Horizontal gene transfer can be mediated by mobile genetic elements such as insertion sequences, transposons, integrative conjugative elements, plasmids and bacteriophages, which are involved in bacterial acquisition and recombination of foreign DNA^34^. Bacteriophages (phages), viruses that infect bacteria, can act as vehicles for horizontal exchange of genetic information, can genetically modify their host by insertion of their DNA into the bacterial genome, and can carry genes that encode new functions or modify existing ones^35^. A recent metagenomic analysis reveals that bacteriophages are reservoirs of antibiotic resistance genes, including genes encoding ATP-binding cassette (ABC) and resistance-nodulation-cell division (RND) proteins, phosphotransferases, β-lactamases and plasmid-mediated quinolone resistance^36^.

In the present study, sequence analysis of one ~104 Kb *bla*_CTX-M-27_-carrying plasmid, named SJ46, revealed that SJ46 was a P1-like bacteriophage bearing an 8,644-bp *bla*_CTX-M-27_-containing region. It was the first time that a *bla*_CTX-M_-type gene was evidenced on a P1-like bacteriophage in 3GC-resistant *Salmonella*. RepA, ParA and ParB of SJ46, are the same as those of a P1-like bacteriophage RCS47 detected in *E. coli* in 2002 in Paris from an adult patient with a urinary infection^11^. RepA of SJ46 only has 42% identity to the RepA of P1 phage (P1 c1-100 Tn9, accession No. AF234172.1) and has no similarities with those of any other replicon proteins from known Inc group plasmids. According to the analysis by Billard-Pomares^11^, the partitioning proteins of RCS47, ParA and ParB, are identical to those of P7 phage, but distantly related to those of P1 phage, which may make it possible to avoid incompatibility with other mobile elements.

The 8.6 Kb inserted element contained a disrupted IS*EcpIB, bla*_CTX-M-27_, IS903D and a putative iron outer membrane receptor gene *iron* and interrupts the methyl-accepting chemotaxis gene *mcp* in Tn1721. A similar structure has been described in the transfer of *bla*_CTX-M-19_ and *bla*_CTX-M-24_ by plasmids in *E. coli* and *Klebsiella pneumonia*, respectively^16^,^37^, which suggested that mobilization of ESBL genes are linked to the IS*Ecp1B*-associated transposition element. The studies of the genetic environments of *bla*_CTX-M_ in the ST131 *E. coli* isolates from hospitals in the Kyoto and Shiga regions of Japan showed that almost all of the CTX-M-27-H30R subgroup had an IS26- ΔIS*Ecp1*- *bla*_CTX-M-27_- ΔIS903D- IS26-like structure, with variation in the length of ΔIS903D^38^,^39^. In our study, the environment of *bla*_CTX-M-27_ had a Tn1721-IS*Ecp1B*-IS903D-ΔTn1721-like structure, and IS*EcpIB* was disrupted by a part of Tn1721 with complete IRL and 5 bp DRs (Fig. 3b), and suggested that *bla*_CTX-M-27_ was transferred to the phage together with IS*Ecp1B* and IS903D from a plasmid by Tn1721 element.

Additionally, the phage-insertion region of SJ46 was also detected in the other six *bla*_CTX-M-27_-carrying plasmids. The PFGE profiles of 3GC-resistant *Salmonella* strains revealed both vertical (clonal) and horizontal spread of the bacteriophage P1-like element among *Salmonella* spp. Moreover, *repL*, which is the lytic replication gene of P1 family phage, was detected in 19.0% of the *Salmonella* isolates. This suggests that this P1-related prophage is common in *Salmonella* from China.

A further limitation of the study is represented by the fact that we did not obtain the sequences of the other six P1 bacteriophage-like plasmids carrying *bla*_CTX-M-27_. However, their lengths expected on the basis of the S1-PFGE results were similar to that of SJ46 and the conserved elements expected in the P1-family bacteriophage were identified.

In conclusion, high rates (15.8%) of third generation cephalosporin resistant *Salmonella*, of which serotypes have been also detected in human infections, were detected in meat from pig carcase from a slaughterhouse in Guangzhou, China. A P1-like bacteriophage SJ46 was found to be related to the transfer of the ESBL gene *bla*_CTX-M-27_ together with a Tn1721 element. Recently, plasmid bearing a *bla*_CTX-M-15_ gene and with chimeric characteristics consisting of pIP1206-like backbone and lysogenized phage P1-like sequences was characterized. It was postulated to have resulted from recombination between an *E. coli* plasmid backbone, a *bla*_CTX-M-15_ bearing region and a lysogenized phage P1-like sequence^40^. The high prevalence of a P1-family phage in *Salmonella* isolates suggest that the abilities of P1-like phages to transfer resistance genes need to be explored more thoroughly. The findings of our study also suggest that plasmid sequencing is necessary to discover the elements involved in the dissemination of antimicrobial resistance genes.

## Materials and Methods

### Sample collection and bacteriological analysis

A total of 1728 pork specimens were obtained between April 2013 and April 2014 from one large-scale slaughterhouse, in Guangdong, China. About 40–50 randomly selected carcasses were sampled weekly before splitting. Samples of 150 g were taken from the inside of two hind legs of each carcass using a sterile knife and placed into a sterile plastic bag. All samples were placed in a low-temperature foam box and dispatched within 3 h to the laboratory. If immediate dispatch was not possible for some reason, samples were stored in a refrigerator at 4 °C until the time of dispatch. About 25 g of meat samples cut into pieces was put into 200 mL buffered peptone water (BPW), which were then incubated at 37 °C for 12 h. One milliliter aliquot of BPW cultures were transfered to 10 mL of selenite cysteine broth and incubated at 37 °C for 24 h. The enriched content was streaked on bismuth sulphite agar and xylose lysine desoxycholate agar and incubated at 37 °C for a further 24 h. Presumptive *Salmonella* colonies were identified with API20E systems (BioMerieux, Beijing, China) and were serotyped using *Salmonella* specific O and H antigens by the slide agglutination test (S&A Company, Bangkok, Thailand).

### Antimicrobial susceptibility testing

Antimicrobial susceptibility testing was conducted using the agar dilution method in accordance with the standards and guidelines described by the Clinical and Laboratory Standards Institute (CLSI). Isolates were tested for sensitivity to cefotaxime, ceftiofur, ceftazidime, ceftriaxone. *Escherichia coli* ATCC 25922 was used as quality control and the susceptibility (or resistance) of each isolate was determined according to the 2015 CLSI recommendations^41^.

### Double disk synergy test

β-lactamase and ESBL-producing isolates were screened according to the double-disk synergy test method recommended by the CLSI combined with antimicrobial disks (cefotaxime 30 μg, cefotaxime-clavulanate 30 μg/10 μg, ceftazidime 30 μg, and ceftazidime-clavulanate 30 μg/10 μg) according to the manufacturer’s instructions (CLSI, 2015). Phenotypic presence of β-lactamases of the isolates was determined by detecting diameter enhancement. If the enhancement value was >5 mm, the isolate was considered to be presumptive β-lactamases producers (CLSI 2015).

### Amplification of β-lactamase-encoding genes

Strains showing resistance to cefotaxime (with MIC ≥ 4 μg/ml) were screened for the presence β-lactamase genes (*bla*_TEM_, *bla*_SHV_, *bla*_OXA_, *bla*_CTX-M_ *bla*_CMY-2_) by PCR using the primers and conditions described previously^42^. The obtained DNA amplicons were submitted to BGI Life Tech Co., Ltd. (Beijing, China) for sequencing and sequences were compared with those included in the GenBank database by using the BLAST algorithm (www.ncbi.nlm.nih.gov) and at www.lahey.org/Studies/ in order to identify specific β-lactamase genes.

### Molecular typing

The genetic relatedness of the isolates was determined by pulsed-field gel electrophoresis (PFGE) using a CHEF-MAPPER System (Bio-Rad Laboratories, Hercules, CA), as previously described^4^. Comparison of PFGE patterns was conducted with BioNumerics software (Applied Maths, Sint-Martens-Latem,Belgium) using the Dice similarity coefficient.

### Determination of replicon types and sizes of plasmids

PCR-based replicon typing (PBRT) was performed on transformants using primers as described previously^43^. The sizes of plasmids carrying *bla*_CTX-M_ was detected by S1-PFGE and southern blot assay. PFGE with S1 nuclease (Takara Biotechnology, Dalian, China) digestion of whole genomic DNA was performed for all transformants. After southern transfer to a Hybond-N+ membrane (GE Healthcare, Little Chalfont, United Kingdom), the plasmids were probed with the *bla*CTX-M gene (DIG High Prime DNA Labeling and Detection Starter Kit I, Roche Applied Science, Mannheim, Germany), as described previously^27^.

### Sequencing and analysis of a *bla*_CTX-M-27_-carrying plasmid

The plasmid from one of the CTX-M-27-carrying isolate J46, was extracted using the Qiagen Plasmid Midi Kit (Qiagen, Germany) and transferred by electroporation in to *E. coli* strain DH5α to yield strain TJ46. Plasmid DNA was purified from TJ46 with the Qiagen Plasmid Midi Kit (Qiagen, Germany) and was sequenced by the Roche 454 Genome Sequencer FLX system. Sequence reads were assembled by SOAP denovo software. The GeneMarKS software (http://topaz.gatech.edu/) was used to identify putative open reading frames^44^. The nucleotide and amino acid sequences were analyzed and compared through BLAST queries against the GenBank database.

### Prevalence investigation of P1-like bacteriophage in *Salmonella* isolates

The presence of a P1-like bacteriophage in *Salmonella* isolated in this study was evaluated by detecting the present of the lytic replication gene of P1 family phage, *repL*, with the primers RepL-fw (5′-CCAATCAACCGTCGTTCGTG-3′) and RepL-rev (5′-TAAGCATATTTCCGCGCTGC-3′)^11^. The PCR method consisted of 30 cycles of denaturation at 94 °C for 30 s, annealing at 55 °C for 30 s and extension at 72 °C for 45 s. This was followed by an additional 10-min extension at 72 °C. The presence of the insertion sequence on the other nontypeable plasmids carrying *bla*_CTX-M-27_ gene was detected using the following primers: IS-fw (AGAATCATCGC CGAAGGGCTGTAACTGGTTTT) and IS-rev (GCGAACATCATCCGTTGCACT CTCTTTGT).

### Statiscal analysis

For statiscal analysis, we carried out Fisher’s exact tests, and *P* values of <0.05 were considered significant.

## Additional Information

**How to cite this article:** Yang, L. *et al*. Characterization of a P1-like bacteriophage carrying CTX-M-27 in *Salmonella spp*. resistant to third generation cephalosporins isolated from pork in China. *Sci. Rep.* **7**, 40710; doi: 10.1038/srep40710 (2017).

**Publisher's note:** Springer Nature remains neutral with regard to jurisdictional claims in published maps and institutional affiliations.

## Supplementary Material

Supplementary Information
