# Corrigendum: Characterization of a P1-like bacteriophage carrying CTX-M-27 in *Salmonella spp*. resistant to third generation cephalosporins isolated from pork in China

**DOI:** 10.1038/srep46728

**Published:** 2017-04-26

**Authors:** Ling Yang, Wan Li, Gui-Ze Jiang, Wen-Hui Zhang, Huan-Zhong Ding, Ya-Hong Liu, Zhen-Ling Zeng, Hong-Xia Jiang

Scientific Reports 7: Article number: 4071010.1038/srep40710; published online: 01 18 2017; updated: 04 26 2017

This Article contains errors in the Materials and Methods section under subheading ‘Prevalence investigation of P1-like bacteriophage in *Salmonella* isolates’, where incorrect primers were quoted.

“The presence of the insertion sequence on the other nontypeable plasmids carrying *bla*_CTX-M-27_ gene was detected using the following primers: IS-fw (AGAATCATCGC CGAAGGGCTGTAACTGGTTTT) and IS-rev (GCGAACATCATCCGTTGCACT CTCTTTGT)”.

should read:

“The presence of the insertion sequence on the other nontypeable plasmids carrying *bla*_CTX-M-27_ gene was detected using the following primers: IR-fw-(GTTGCTGGCTGACGCCTATGAAG) and IR-rev-(ATGTTTGCCATTTCATAGGGGAG)”.

