# Supplementary material for: Characterization of a P1-like bacteriophage carrying CTX-M-27 in Salmonella spp. resistant to third generation cephalosporins isolated from pork in China
Source: Sci Rep. 2017 Jan 18;7:40710. doi: 10.1038/srep40710 (PMC5241659; doi:10.1038/srep40710)
Supplement: Supplementary Information [file srep40710-s1.pdf]

Supplementary Data

Characterization of a P1-like bacteriophage carrying CTX-M-27 in *Salmonella spp.* resistant to third generation cephalosporins isolated from pork in China

Ling Yang[1,2†], Wan Li[1†], Gui-Ze Jiang[1], Wen-Hui Zhang[1], Huan-Zhong Ding[2], Ya-Hong Liu[1], Zhen-Ling Zeng[2]*and Hong-Xia Jiang[1]*

College of Veterinary Medicine, [1]National Risk Assessment laboratory for antimicrobial resistance of animal original bacteria, [2]Guangdong Provincial Key Laboratory of Veterinary Pharmaceutics Development and Safety Evaluation, South China Agricultural University (SCAU), Guangzhou 510642, China;

† Equal contribution

*Corresponding author

Email: hxjiang@scau.edu.cn;

Tel: +86 20 85283934

Fax: +86 20 85284896

Email: zlzeng@scau.edu.cn

Tel: +86 20 85281204

Fax: +86 20 85284899

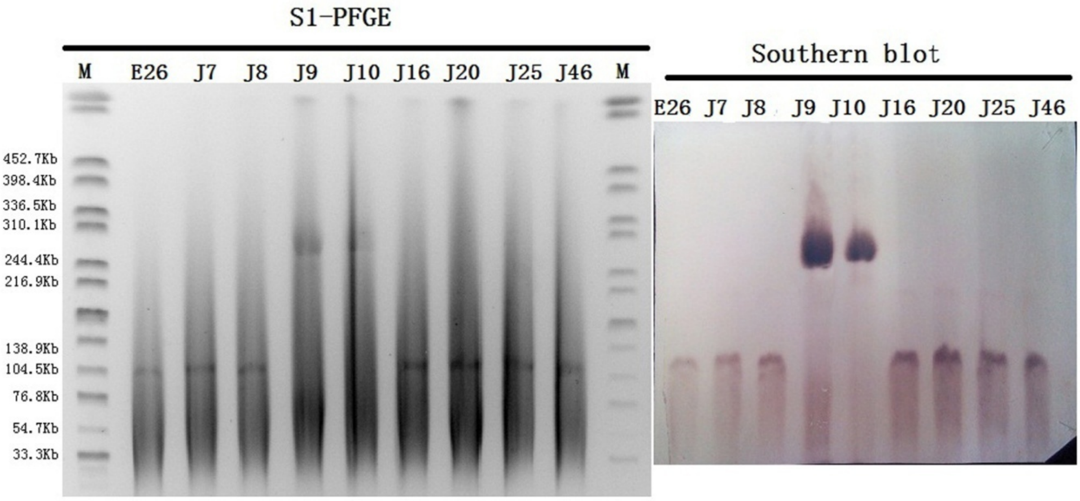

Figure S1. Results of S1-PFGE and southern blot assay for nine *bla*$_{CTX-M-27}$-containing transformants

Table S1. Features of predicted ORFs in SJ46

ORF	ORF position	Coding strand	Length (aa)	Best GenBank match , aa, Accession No.	E value, aa identity	conserved domains	Putative functional assignment, relevant reference(s)
**1**	245..529	-	94	Uhr [Enterobacteria phage P1], 94aa, YP_006535.1	6e-65, 94/94(100%)	-	Unknown protein function
**2**	804..983	+	59	UpfC [Enterobacteria phage P1], 59aa, YP_006534.1	2e-34, 59/59(100%)	-	Unknown protein function
**3**	992..1780	+	262	UpfB [Escherichia coli 2362-75] , 262aa, EFR18857.1	0.0, 262/262(100%)	-	Unknown protein function
**4**	1820..2242	+	140	PpfA [Enterobacteria phage P1, 140aa, YP_006532.1	1e-97, 139/140(99%)	-	Possible periplasmic function
**5**	2277..2408	+	43	membrane lipoprotein precursor Mlp [Escherichia phage RCS47], 43aa, CDN90812.1	2e-23, 43/43(100%)	-	Membrane lipoprotein precursor (by homology)
**6**	2420..2812	+	130	UpfA [Enterobacteria phage P1], 130aa, YP_006530.1	3e-89, 128/130(98%)	-	Unknown protein function
**7**	3133..4031	+	294	replication protein RepA [Escherichia phage RCS47], 294aa, CDN90810.1	0.0, 294/294(100%)	-	Plasmid replication initiated by chaperone-activated monomers at oriR; represses own synthesis; binds at iterated sites (incC and incA) and can handcuff them, controling plasmid copy number
**8**	4324..5007	+	227	hypothetical protein EC54115_07577 [Escherichia coli 541-15] , 269aa, EIL56554.1	2e-170, 227/227(100%)	T5orf172; GIY-YIG_SF superfamily	Unknown protein function
**9**	5302..6498	+	398	partition protein ParA [Escherichia phage RCS47], 398aa, CDN90808.1	0.0, 398/398(100%)	BcsQ; ParA; P-loop_NTPase superfamily	Plasmid partitioning; weak ATPase, binds to parO repressing transcription, binds to ParB-parS

							partition complex
**10**	6515..7516	+	333	ParB [Enterobacteria phage P1], 333aa, YP_006527.1	0.0, 332/333(99%)	ParB superfamily; ParBc; Spo0J; parB_part	Plasmid partitioning; binds to parS, enhances ParA-mediated repression of par operon and ATPase of ParA; can spread over DNA flanking parS, silencing gene expression; pairs parS loci
**11**	7742..9448	+	568	Gp23 [Enterobacteria phage P1], 568aa, YP_006526.1	0.0, 567/568(99%)	-	Major head protein, present in P1 heads in full-length (62 kDa) and truncated (44 kDa) forms
**12**	9509..11098	+	529	hypothetical protein AF33_04663, Gp22 [Escherichia coli CHS 77]; 529aa, KDG50404.1	0.0, 526/529(99%)	-	Sheath protein
**13**	11108..11923	+	271	tail tube protein Gp21 [Escherichia phage RCS47], 271aa, CDN90804.1	6e-175, 262/271(97%)	-	Baseplate or tail tube
**14**	11959..12540	+	193	PmgG [Enterobacteria phage P1], 193aa, YP_006523.1	3e-136, 191/193(99%)	-	Putative morphogenetic function
**15**	12552..13061	+	169	BplB [Enterobacteria phage P1], 169aa, YP_006522.1	1e-122, 168/169(99%)	-	Putative baseplate structural protein
**16**	13197..13829	+	210	hypothetical protein Y979_25865 (plasmid) [Escherichia coli str. Sanji], 210aa, AMU85563.1	1e-151, 210/210(100%)	-	Unknown protein function
**17**	14009..14260	-	83	membrane protein [Escherichia coli], 81aa, AHM43618.1|	6e-46, 73/81(90%)	-	Membrane protein
**18**	14320..14835	-	171	attachment protein [Escherichia coli], 171aa, AHM43619.1	1e-122, 171/171(100%)	ompX; Ail_Lom; OM_channels superfamily	Unknown protein function
**19**	14931..15773	-	280	RepL [Enterobacteria phage P7] , 281aa,	0.0,	-	Lytic replication, initiates replication

				CDN90799.1	261/281(93%)		at oriL (within repL)
**20**	15803..16603	-	266	KilA [Enterobacteria phage P7], 266aa, AAQ07519.1	0.0, 266/266(100%)	-	Unknown,expression can kill host
**21**	16768..17814	-	348	Antirepressor protein, Ant1[Enterobacteria phage P7], 348aa, AAQ07516.1	0.0, 344/348(99%)	-	Antagonism of C1 repression; forms complex with Ant2
**22**	18643..19164	+	173	Upf 50.5 [Enterobacteria phage P7], 173aa, AAQ07514.1	1e-118, 173/173(100%)	Radical SAM superfamily	Outer membrane lipoprotein of unknown function
**23**	19168..19707	+	179	Upf 50.0 [Enterobacteria phage P7], 179aa, AAQ07513.1	2e-118, 179/179(100%)	phosphodiesterase; Provisional (PRK12704); Protein of unknown function (DUF3584)	Conserved membrane protein of unknown function
**24**	19836..20354	+	172	PmgC [Escherichia phage RCS47], 188aa, CDN90792.1	5e-118, 169/172(98%)	-	Putative morphogenetic protein
**25**	20365..20976	+	203	phage major tail tube protein, TubB [Escherichia coli UMNF18], 203aa, AEJ60191.1	8e-146, 201/203(99%)	-	Phage major tail tube protein
**26**	20991..21827	+	293	PmgB [Escherichia coli UMNF18], 293aa, AEJ60190.1	0.0, 292/293(99%)	-	Phage morphogenetic protein
**27**	21954..25367	+	1137	Sit [Enterobacteria phage P1], 1140aa, YP_006507.1	0.0, 993/1140(87%)	Lytic Transglycosylase (LT) and Goose Egg White Lysozyme (GEWL) domain; Transglycosylase SLT domain; Secretion system effector C (SseC) like family; internal virion protein D	Structural injection transglycosylase; putative tail tube “ruler”

**28**	25367..25763	+	118	PmgA [Enterobacteria phage P1], 118aa, YP_006506.1	2e-81, 118/118(100%)	-	Putative morphogenetic protein
**29**	25720..27153	+	477	BplA [Enterobacteria phage P1], 477aa, YP_006505.1	0.0, 477/477(100%)	-	Putative baseplate structural protein
**30**	27153..27989	+	278	Gp16 [Escherichia coli 2362-75], 278aa, EFR18935.1	0.0, 278/278(100%)		Putative tail tube protein
**31**	28068..28502	+	144	GpR [Enterobacteria phage P7], 144aa, AAQ07505.1	2e-101, 144/144(100%)	-	Structure or assembly of tail fibers
**32**	28514..30856	+	780	major tail fiber protein, partial [Salmonella enterica subsp. enterica serovar Newport str. JS09102], 457aa, EMG75401.1	0.0, 457/457(100%)	Phage tail fibre repeat; Phage-related tail fibre protein	Tail fiber protein
**33**	30858..31625	+	135	Fels-2 prophage Tfa [Salmonella enterica subsp. enterica serovar Rissen str. 150], 135aa, ELX20892.1	9e-96, 135/135(100%)	Caudo-TAP super family	Caudovirales tail fibre assembly protein
**34**	31269..31856	-	195	phage tail-fiber asembly protein [Salmonella enterica subsp. enterica serovar Agona str. SH08SF124], 195aa, ELP15074.1	7e-143, 195/195(100%)	Caudo-TAP super family	Phage tail-fiber asembly protein
**35**	31856..33181	-	441	phage tail protein [Salmonella enterica subsp. enterica serovar Infantis], 554aa, KNK93741.1	0.0, 417/440(95%)	Phage-related tail fibre protein (COG5301)	Phage tail protein
**36**	33176..33757	+	193	site-specific recombinase, DNA invertase Pin [Salmonella enterica subsp. enterica serovar Newport str. JS09102], 193aa, EMG75300.1	7e-140, 193/193(100%)	Serine Recombinase (SR) family; ; mpi; PinE; HTH_Hin_like superfamily	Site-specific recombinase

**37**	33889..34113	-	74	DNA-invertase [Salmonella enterica subsp. enterica serovar Newport str. JS09102], 74aa, EMG75301.1	1e-47, 74/74(100%)	Serine Recombinase (SR) family; Resolvase; N terminal domain; PinE; mpi	DNA-invertase
**38**	34178..34522	+	114	hypothetical protein G209_23302 [Salmonella enterica subsp. enterica serovar Newport str. JS09102], 114aa, EMG75302.1	6e-79, 114/114(100%)	-	Unknown protein function
**39**	34590..34919	+	109	LydA [Enterobacteria phage P1], 109aa, YP_006497.1	9e-71, 109/109(100%)	-	Lysis determinant; putative holin (by homology)
**40**	34916..35359	+	147	LydB [Enterobacteria phage P7], 147aa, AAQ07501.1	2e-106, 147/147(100%)	-	Lysis determinant; prevents premature lysis, LydA antagonist
**41**	35346..35948	+	200	conserved protein of unknown function Hdf [Escherichia phage RCS47], 200aa, CDN90775.1	1e-137, 198/200(99%)	-	Uncertain; homolog to DarA fragment; defect possibly causes *gta*(generalized transduction affected) phenotype
**42**	35950..37869	+	639	DarA [Escherichia phage RCS47], 639aa, CDN90774.1	0.0, 633/639(99%)	Prefoldin subunit; SH3 domain protein; PRK09039; Chromosome segregation ATPase；Spc7 kinetochore protein	Defends against restriction by type I Res endonuclease and enables DarB to function; internal head protein processed by Pro
**43**	37866..38231	+	121	DdrA [Salmonella enterica subsp. enterica serovar Derby], 121aa, KNQ83755.1	2e-80, 120/121(99%)	-	Uncertain; product of gene downstream of *darA*; defect possibly alters P1B:P1S:P1M ratios; *vad* (viral architecture determinant)
**44**	38271..39479	-	402	transposase [Escherichia coli], 402aa, WP_024183914.1	0.0, 389/402(97%)	HTH_OrfB_IS605;InsQ; OrfB_IS605 superfamily; OrfB_Zn_ribbon	Transposase
**45**	39579..42566	+	995	DdrB [Escherichia coli], 995aa,	0.0,	-	Unknown; product of the second

				KYV78578.1	995/995(100%)		gene downstream of darA; possible protease or response regulator
**46**	42556..42867	+	311	putative repressor protein Hxr [Escherichia phage RCS47], 311aa, CDN90770.1	5e-67, 103/103(100%)	-	Function proposed based on presence of conserved amino acid motif, structural f
**47**	42897..43679	-	260	conserved protein of unknown function, IsaA [Escherichia phage RCS47], 260aa, CDN90768.1	0.0, 259/260(99%)	-	Homolog of xre; possible repressor protein
**48**	43686..44531	-	281	PF11195 family protein [Escherichia coli JB1-95], 281aa, EIH87607.1	0.0, 280/281(99%)	-	Protein of unknown function
**49**	44561..45049	-	162	Single-stranded DNA-binding protein, Ssb [Enterobacteria phage P1], 162aa, YP_006485.1	2e-116, 162/162(100%)	SSB; RPA_2b-aaRSs_OBF_like superfamily; PHA01740 superfamily	Single-stranded DNA binding protein; can suppress E. coli ssb
**50**	45132..45776	+	214	phage lysozyme, Lyz[Escherichia coli 907713], 214aa, ESA89767.1	3e-159, 214/214(100%)	lysozyme_like superfamily	Lysozyme
**51**	45769..46020	+	83	LydD, putative holin [Enterobacteria phage P1], 84aa, YP_006483.1	2e-50, 81/83(98%)	-	Lysis determinant D, putative holin
**52**	46068..47087	-	339	putative head processing protein, Pro[Salmonella enterica subsp. enterica serovar Newport str. JS09102], 339aa, EMG75315.1	0,0, 339/339(100%)	NTP-PPase superfamily	Putative head processing protease and kinase; required for head morphogenesis and maturation of DarA precursor protein
**53**	47080..48789	-	569	putative portal protein, Prt[Salmonella enterica subsp. enterica serovar Newport str. JS09102], 569aa, EMG75316.1	0.0, 569/569(100%)	-	Portal protein (by similarity)
**54**	48880..53361	+	1493	helicase, DarB[Salmonella enterica subsp. enterica serovar Derby], 2255aa, KNQ83765.1	0.0, 1486/1493(99 %)	DEXDc; ResIII; dnd_restrict_1; Methyltransf_26; HsdM; N-6_Mtase; SNF2_N;	Defense (in cis) against a subset of type I Res enzymes, e.g. EcoB, EcoK; putative DNA methyltransferase and DNA helicase;

						HepA	may methylate DNA during injection
**55**	53542..55632	+	696	helicase conserved C-terminal domain protein [Escherichia coli DEC6A], 2218aa, EHV49330.1	0.0, 689/696(99%)	heterocyst_DevB	Defense against restriction protein
**56**	55666..56106	+	146	putative protein Ulx [Escherichia phage RCS47], 146aa, KGM68784.1	1e-101, 146/146(100%)	-	Unknown protein function
**57**	56103..56351	+	82	Lxc [Enterobacteria phage P7], 82aa, AAQ07487.1	9e-55, 82/82(100%)	-	Modulator of C1 action
**58**	56389..57510	-	373	GTP pyrophosphokinase [Salmonella enterica subsp. enterica serovar Newport str. JS09102], 373aa, EMG75319.1	0.0, 373/373(100%)	NT_Rel-Spo_like; RelA_SpoT; YjbM; Glyco_hydro_15	Lowers expression of c1, enhances binding of C1 to its operators
**59**	57623..58264	-	213	Maturation control protein, Mat[Escherichia phage RCS47], 213aa, CDN90745.1	3e-154, 209/213(98%)	-	Particle maturation
**60**	58513..61479	-	988	transposase for transposon Tn1721 [Klebsiella pneumoniae subsp. pneumoniae HS11286], 988aa, YP_005220836.1	0.0, 988/988(100%)	DDE_Tnp_Tn3; COG4644; DUF4158	Transposase for transposon
**61**	61483..61857	-	124	DNA resolvase, tnpR [Escherichia coli], 153aa, WP_061089326.1	8e-83, 124/124(100%)	SR_ResInv; HTH_Hin_like; Resolvase; HTH_7; PinE; mpi	DNA resolvase
**62**	61861..62199	+	112	hypothetical protein [Escherichia coli IS5], 112aa, CDK50280.1	9e-75, 112/112(100%)	-	Unknown protein function
**63**	62353..62982	+	209	methyl-accepting chemotaxis protein [Escherichia coli], 209aa, WP_013188478.1	2e-153, 208/209(99%)	PAS; PAS_3; PRK13558	Methyl-accepting chemotaxis protein
**64**	62957..63982	-	341	iron outer membrane receptor, IroN (plasmid) [Escherichia coli] 341aa, AKI06962.1	0.0, 341/341(100%)	ligand_gated_channel; TonB_dep_Rec; CirA; TonB-siderophor;	

						PRK09840	
**65**	64111..65079	-	322	transposase InsC for insertion sequence IS903 [Klebsiella pneumoniae subsp. pneumoniae HS11286], 322aa, YP_005224844.1	0.0, 322/322(100%)	DDE_Tnp_1_5; DDE_Tnp_1	Transposase
**66**	65114..65989	-	291	class A extended-spectrum beta-lactamase CTX-M-27 [Escherichia coli], 291aa, WP_032277257.1	0.0, 291/291(100%)	Beta-lactamase2; Transpeptidase superfamily;	Extended-spectrum beta-lactamase
**67**	66239..66496	-	85	ISEcp1B transposase, partial [Proteus mirabilis], 347aa, AGE10672.1	5e-53, 85/85(100%)	DDE_Tnp_1_4	Transposase
**68**	66576..66923	+	115	Methyl-accepting chemotaxis protein (MCP) signaling domain [Escherichia coli], 115aa, CTV83025.1	4e-75, 115/115(100%)	MCP_signal	Methyl-accepting chemotaxis protein
**69**	67071..67652	-	193	Ref [Enterobacteria phage P7], 216aa, AAQ07480.1	7e-124, 174/187(93%)	-	Recombination enhancement function
**70**	67900..68211	-	103	lysogeny establishment protein, C8 [Salmonella enterica subsp. enterica serovar Infantis], 103aa, KNN15215.1	8e-68, 103/103(100%)	-	Establishment of lysogeny?
**71**	68262..69293	-	343	Cre [Enterobacteria phage P1], 343aa, YP_006472.1	0.0, 342/343(99%)	INT_Cre_C; Phage_integrase; Phage_int_SAM_4; recomb_XerC; XerD	Cyclization recombinase
**72**	69301..69522	-	73	Cra [Enterobacteria phage P1], 73aa, YP_006471.1	6e-47, 72/73(99%)	-	Putative cre associated function
**73**	70127..70336	+	69	Coi, C1 repressor inactivator [Enterobacteria phage P1], 69aa, YP_006578.1	4e-43, 69/69(100%)	-	C1 (reversible) inactivator; forms 1:1 complex with C1
**74**	70447..71298	+	283	phage repressor protein , C1[Enterobacteria phage phiW39], 283aa,	0.0, 283/283(100%)	-	Primary repressor of lytic function

				AAV84927.1			
**75**	71323..72807	-	494	terminase B protein PacB [Escherichia phage RCS47], 494aa, CDN90859.1	0.0, 492/494(99%)	-	DNA packaging; cuts at pac together with PacA
**76**	72807..74000	-	397	Terminase A protein PacA [Escherichia phage RCS47], 397aa, CDN90858.1	0.0, 392/397(99%)	-	DNA packaging; cuts at pac together with PacB
**77**	74087..74509	-	140	late promoter-activating protein (Gp10) [Escherichia coli M605], 150aa, EGI13364.1	2e-98, 140/140(100%)	-	Late promoter activator
**78**	74628..75671	-	347	PdcB [Enterobacteria phage P1], 347aa, YP_006573.1	0.0, 343/347(99%)	DUF968 superfamily	Unknown; post-doc
**79**	75699..75878	-	59	PdcA [Escherichia coli ISC7], 59aa, CDL28025.1	2e-33, 59/59(100%)	-	Unknown; post-doc
**80**	75883..76263	-	126	Doc [Enterobacteria phage P1], 126aa, YP_006571.1	1e-85, 126/126(100%)	DOC_P1; Doc; Fic	Death on curing; toxin of P1 addiction system; reversible inhibitor of protein synthesis; corepressor of addiction operon
**81**	76263..76484	-	73	Phd [Enterobacteria phage P1]; 73aa, YP_006570.1	9e-45, 73/73(100%)	PhdYeFM_antitox; Phd; phd_fam	antitoxin of P1 addiction system; Prevent host death protein
**82**	76557..76946	-	129	DNA repair protein HumD [Enterobacteria phage P1], 129aa, YP_006569.1	1e-89, 128/129(99%)	S24_LexA-like; PRK10276; LexA;	Homolog of UmuD' subunit of E. coli repair protein DNA polymerase V (UmuD'-UmuC); complements mutant UmuD'
**83**	77121..77705	+	194	Upf94.2 [Enterobacteria phage P7], 194aa, AAQ07574.1	2e-142, 192/194(99%)	-	Unknown protein function
**84**	77706..78062	+	118	hypothetical protein AEW06_01065 [Salmonella enterica subsp. enterica serovar Derby], 118aa, KNQ83785.1	4e-81, 118/118(100%)	-	Unknown protein function
**85**	78925..79062	+	45	hypothetical protein EC40522_B0049	8e-22,	-	Unknown protein function

				[Escherichia coli 4.0522], 45aa, EIH76026.1	45/45(100%)		
**86**	79138..79500	-	120	PmgV [Enterobacteria phage P1], 120aa,	8e-76, 116/120(97%)	-	Putative morphogenetic function
**87**	79497..80429	-	310	hypothetical protein G209_23492 [Salmonella enterica subsp. enterica serovar Newport str. JS09102], 310aa, EMG75338.1	0.0, 310/310(100%)	-	Unknown protein function
**88**	80411..80785	-	124	hypothetical protein G209_23497 [Salmonella enterica subsp. enterica serovar Newport str. JS09102], 124aa, EMG75339.1	2e-87, 124/124(100%)	-	Unknown protein function
**89**	80792..81052	-	86	hypothetical protein G209_23502 [Salmonella enterica subsp. enterica serovar Newport str. JS09102], 86aa, EMG75340.1	9e-58, 86/86(100%)	-	Unknown protein function
**90**	81093..81215	-	40	hypothetical protein SEEPO729_20495 [Salmonella enterica subsp. enterica serovar Pomona str. ATCC 10729] , 40aa, EHN41314.1	1e-19, 40/40(100%)	-	Unknown protein function
**91**	81264..81497	-	77	hypothetical protein G209_23507 [Salmonella enterica subsp. enterica serovar Newport str. JS09102], 77aa, EMG75341.1	3e-48, 77/77(100%)	-	Unknown protein function
**92**	81574..81834	-	86	hypothetical protein G209_23512 [Salmonella enterica subsp. enterica serovar Newport str. JS09102], 85aa, EMG75342.1	3e-55, 85/85(100%)	-	Unknown protein function
**93**	81831..82691	-	286	Eaa protein [Salmonella enterica subsp.	0.0,	DUF551 superfamily	Phage protein,

				enterica serovar Newport str. JS09102], 286aa, EMG75343.1	286/286(100%)		
**94**	82693..83373	-	226	ead/Ea22-like family protein [Escherichia coli 2-474-04_S1_C1], 226aa, KDY77502.1	1e-164, 223/226(99%)	Ead_Ea22 superfamily	Unknown protein function
**95**	83370..84014	-	214	hypothetical protein G209_23006 [Salmonella enterica subsp. enterica serovar Newport str. JS09102], 214aa, EMG75346.1 2-Upf87.6 [Enterobacteria phage P7], AAQ07562.1	9e-162, 214/214(100%)	-	Unknown protein function; encodes putative tyrosine protein phosphatase
**96**	84007..84267	-	86	Upf87.4 [Enterobacteria phage P7], 86aa, AAQ07561.1	6e-58, 86/86(100%)	-	Unknown protein function
**97**	84260..84841	-	193	Upf86.8 [Enterobacteria phage P7], 193aa, AAQ07560.1	6e-141, 192/193(99%)	ASCH_yqfb_like	Unknown protein function
**98**	84851..84985	-	44	putative membrane protein [Escherichia coli 2362-75], 44aa, EFR18883.1	2e-23, 44/44(100%)	-	Putative membrane protein
**99**	85036..85542	-	168	putative morphogenetic protein [Salmonella enterica subsp. enterica serovar Newport str. JS09102],	2e-122, 168/168(100%)	-	Putative membrane protein
**100**	85615..86877	-	420	putative morphogenetic protein , PmgS[Salmonella enterica subsp. enterica serovar Newport str. JS09102], 168aa, EMG75351.1	0.0, 420/420(100%)	Phage_GP20; SMC_prok_B; PRK02224; SCP-1; Smc	Putative morphogenetic protein
**101**	87179..87880	-	233	putative morphogenic function protein PmgQ [Escherichia phage RCS47], 233aa, CDN90836.1	1e-171, 232/233(99%)	-	Head morphogenesis
**102**	87877..88569	-	230	Ppp [Enterobacteria phage P7], 230aa,	8e-172,	MPP_PrpA_PrpB; pphA;	Protein phosphatase

				AAQ07555.1	229/230(99%)	Metallophos; apaH; PP2Ac	
**103**	88551..89087	-	178	PmgP [Escherichia coli O10:K5(L):H4 str. ATCC 23506], 208aa, CCP95118.1	3e-129, 176/178(99%)	-	Putative norphogenetic protein
**104**	89075..89575	-	166	putative morphogenic function protein PmgO [Escherichia phage RCS47], 220aa, CDN90833.1	9e-119, 165/166(99%)	-	Prophage functions
**105**	89679..89834	-	51	PmgN [Enterobacteria phage P1], 51aa, YP_006553.1	6e-29, 51/51(100%)	-	Putative norphogenetic protein
**106**	89901..90479	-	192	pmgM [Escherichia coli 5905], 192aa, EKH93593.1	2e-139, 189/192(98%)	VRR_NUC superfamily	Norphogenetic protein
**107**	90482..90727	-	81	PmgL [Enterobacteria phage P1], 81aa, YP_006551.1	3e-53, 80/81(99%)	DiS_P_DiS superfamily	putative morphogenetic function
**108**	90874..91251	+	125	gp26 [Enterobacteria phage P1], 125aa, YP_006550.1	6e-88, 125/125(100%)	-	Putative baseplate protein
**109**	91261..92478	+	405	gp25 [Enterobacteria phage P1], 405aa, YP_006549.1	0.0, 403/405(99%)	PTZ00121	Phage tail protein
**110**	92482..93210	+	242	gp7 [Enterobacteria phage P1], 242aa, YP_006548.1	3e-178, 242/242(100%)	-	Phage tail protein
**111**	93197..93982	+	261	gp24 [Enterobacteria phage P1], 261, YP_006547.1	0.0, 260/261(99%)	-	Baseplate
**112**	93984..95000	+	338	Upf77.7 [Escherichia coli STEC_C165-02], 338aa, EGW72531.1	0.0, 338/338(100%)	-	Phage tail length tape measure protein [Escherichia coli]
**113**	94993..95625	+	210	Upf76.8 [Enterobacteria phage P7], 210aa, AAQ07544.1	4e-149, 209/210(99%)	-	Phage-related baseplate assembly protein
**114**	95672..96670	-	332	hypothetical protein SEEPO729_20365 [Salmonella enterica subsp. enterica serovar Pomona str. ATCC 10729], 332aa, EHN41288.1	0.0, 332/332(100%)	-	Unknown protein function
**115**	96670..98034	-	454	Ban [Enterobacteria phage P1], 454aa,	0.0,	DnaB_C; P-loop_NTPase	Replicative DNA helicase

				YP_006543.1	451/454(99%)	superfamily	
**116**	98395..98502	-	35	hypothetical protein SEEPO729_20355 [Salmonella enterica subsp. enterica serovar Pomona str. ATCC 10729], 35aa, EHN41286.1	1e-10, 35/35(100%)	-	Possibly tellurite or colicin resistance or inhibition of cell division accessory protein
**117**	98502..98666	-	54	TciB [Enterobacteria phage P1], 54aa, YP_006541.1	1e-16, 52/54(96%)	DUF3927 superfamily	Tellurite or colicin resistance or inhibition of cell division accessory protein (by homology)"
**118**	98666..99091	-	141	TciA [Enterobacteria phage P1], 141aa, YP_006540.1	1e-97, 141/141(100%)	TerB	Tellurite or colicin resistance or inhibition of cell division (by homology
**119**	99324..99455	-	43	hypothetical protein PPECC9_43920 [Escherichia coli PCN009], 43aa, OAF89450.1	7e-23, 43/43(100%)	-	Unknown protein function
**120**	99455..99874	-	139	No similar protein	-	-	Unknown protein function
**121**	99890..100207	+	105	hypothetical protein [Escherichia coli], 105aa, WP_000711133.1	2e-72, 104/105(99%)	-	Unknown protein function
**122**	100372..102795	-	807	DMT family permease ,Dmt[Escherichia coli], 807aa, WP_000660975.1	0.0, 797/807(99%)	Dcm superfamily; AdoMet_MTases superfamily	Predicted bifunctional DNA methyltransferase; methylates A's in GATC sequences, and (by homology) probably C's at unknown

a NCBI Database of non-redundant protein sequences was searched using Blastp (protein-protein BLAST)
